# Neural Stem Cells and Its Derivatives as a New Material for Melanin Inhibition

**DOI:** 10.3390/ijms19010036

**Published:** 2017-12-22

**Authors:** Insik Hwang, Sunghoi Hong

**Affiliations:** 1 School of Biosystem and Biomedical Science, College of Health Science, Korea University, 22 Gil Inchon-ro, Seongbuk-gu, Seoul 02855, Korea; dlstlr@korea.ac.kr; 2 Department of Public Health Sciences, Korea University Graduate School, 22 Gil Inchon-ro, Seongbuk-gu, Seoul 02855, Korea; 3 Department of Integrated Biomedical and Life Science, Korea University Graduate School, 22 Gil Inchon-ro, Seongbuk-gu, Seoul 02855, Korea

**Keywords:** melanin, depigmentation, neural stem cells (NSCs), conditioned medium (CM), secreted factors

## Abstract

The pigment molecule, melanin, is produced from melanosomes of melanocytes through melanogenesis, which is a complex process involving a combination of chemical and enzymatically catalyzed reactions. The synthesis of melanin is primarily influenced by tyrosinase (TYR), which has attracted interest as a target molecule for the regulation of pigmentation or depigmentation in skin. Thus, direct inhibitors of TYR activity have been sought from various natural and synthetic materials. However, due to issues with these inhibitors, such as weak or permanent ability for depigmentation, allergy, irritant dermatitis and rapid oxidation, in vitro and in vivo, the development of new materials that inhibit melanin production is essential. A conditioned medium (CM) derived from stem cells contains many cell-secreted factors, such as cytokines, chemokines, growth factors and extracellular vesicles including exosomes. In addition, the secreted factors could negatively regulate melanin production through stimulation of a microenvironment of skin tissue in a paracrine manner, which allows the neural stem cell CM to be explored as a new material for skin depigmentation. In this review, we will summarize the current knowledge regulating depigmentation, and discuss the potential of neural stem cells and their derivatives, as a new material for skin depigmentation.

## 1. Introduction

**Figure 1:**
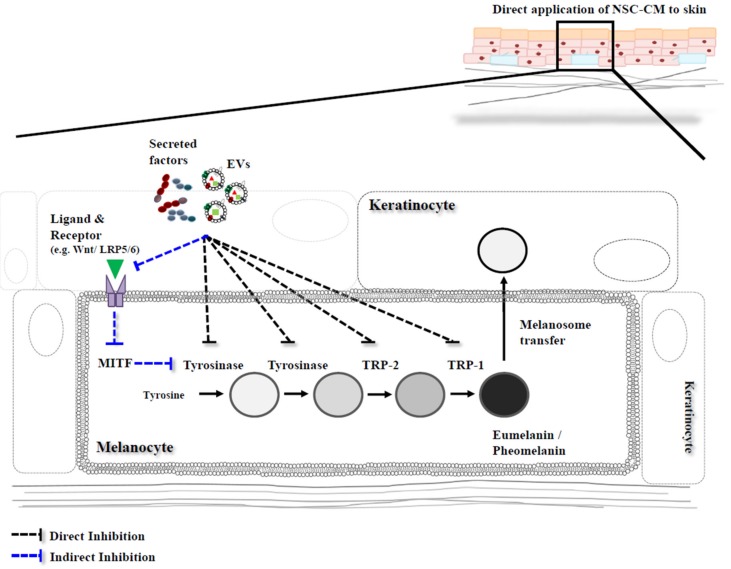
A schematic strategy inhibiting the pathways of melanin synthesis. The secreted factors and extracellular vesicles (EVs) including exosomes and microvesicles lead to multiple actions in a paracrine manner, which may be directly and/or indirectly involved in inhibiting the pathways of melanin synthesis. One is that the secreted factors and EVs directly regulate the expression of the melanogenic enzymes, the other one is that the secreted factors and EVs indirectly regulate the melanogenic genes via regulation of molecular signaling genes and subsequently *MITF* gene expression. NSC-CM, neural stem cells-conditioned medium. TRP-1, tyrosinase related protein-1 TRP-2, tyrosinase related protein-2.

Melanin is produced in epidermal melanocytes by melanogenesis, a process involving various chemical and enzymatically catalyzed reactions [1]. Tyrosinase (TYR) is essential for melanin formation. This copper-containing enzyme can catalyze three different reactions involved in melanin biosynthesis: the hydroxylation of tyrosine to 3,4-dihydroxyphenylalanoine (DOPA); the oxidation of DOPA to DOPAquinone; and the oxidation of 5,6-dihydroxyindole (DHI) to indole-quinone. The first step is the rate-limiting step in melanin synthesis because the remainder of the reaction sequence can proceed spontaneously at physiological pH [2,3]. After DOPA is formed, it undergoes auto-oxidation and/or is catalyzed by tyrosinase-related protein-2/dopachrome tautomerase (TRP-2/DCT) and tyrosinase-related protein-1 (TRP-1) to DOPAquinone [4,5]. In this process, two types of melanin are synthesized within melanosomes: a dark brown-black insoluble eumelanin and a light red-yellow soluble pheomelanin [6,7] (Figure 1). Although TYR, TRP-1 and TRP-2 are all involved in melanogenesis, TYR plays the most critical roles in melanin formation (i.e., rate-limiting step in the process) [8,9].

Microphthalmia-associated transcription factor (*MITF*) also plays a key role in regulating melanin synthesis pathways and all of the melanogenic enzymes are transcriptional targets of *MITF*. The promoters of tyrosinase (*TYR)*, tyrosinase related protein-1 (*TRP-1*) and tyrosinase related protein-2 (*TRP-2)* genes, possess an *MITF* binding motif (5′-CATGTG-3′). During melanocyte development, *MITF* directly regulates the expression of these melanogenic enzymes. The *MITF* gene and protein are not only initiated by a number of signaling pathways but are also regulated by transcriptional and post-transcriptional pathways. Furthermore, several transcription factors, Paired box protein (PAX3), cyclic adenosine monophosphate response element-binding (CREB), SRY-related HMG-box (SOX10) and lymphoid-enhancing factor/T-cell factors (LEF/TCF), bind to the *MITF* promoter and regulate its transcription. The melanocortin-1 receptor (MC1R) (ligand; α-melanocyte-stimulating hormone, α-MSH) involves the activation of cyclic adenosine monophosphate (AMP) and cyclic adenosine monophosphate response element-binding (CREB), which regulates *MITF*. Along with the MC1R pathway, the tyrosine kinase receptor c-kit (ligand; stem cell factor, SCF) signaling pathway, stimulates mitogen-activated protein kinase (MAPK) and modulates *MITF*. Additionally, the Wnt/β-catenin pathway, Frizzled proteins and lipoprotein-receptor-related proteins 5 and 6 (LRP5/6) (ligand; Wnt), regulate transcription of *MITF* through interactions with LEF/TCF, which stabilize cytoplasmic β-catenin and transport it into the nucleus.

Melanin has a beneficial role in protecting human skin from harmful effects of ultraviolet (UV) radiation, while an excessive melanin production causes dermatological problems, such as freckles, age spots (solar lentigo) [10] and melasma (skin cancer) [11]. In the context of preventing hyperpigmentation, inhibition of TYR is the main motivation for cosmetics or skin whitening because tyrosinase is a crucial enzyme in melanin synthesis [12]. Most tyrosinase inhibitors directly inhibit tyrosinase activity. Tyrosinase inhibitors, such as hydroquinone [13,14], arbutin [15], deoxyarbutin [16], kojic acid [17], azelaic acid [18], aloesin [19], licorice [20], L-ascorbic acid [21], ellagic acid [22], tranexamic acid [23], and various phenolic compounds, have been used to inhibit melanin synthesis However, certain disadvantages have been reported with these compounds. For instance, hydroquinone causes permanent leukemia, skin irritation, contact dermatitis, loss of skin elasticity and exogenous ochronosis [24,25]. The natural form of arbutin can release hydroquinone, which is catabolized to benzene metabolites and has potential toxicity [26]. The use of kojic acid in cosmetics, is limited because of carcinogenicity, allergic reactions (e.g., dermatitis and sensitization), and storage instability [27]. L-ascorbic acid displays chemically instability and has a tendency to rapidly oxidize in aqueous solution (Table 1).

Recently, it has been reported that the various factors secreted from different types of stem cell such as mesenchymal stem cells (MSCs) or neural stem cells (NSCs) regulate melanin production [28,29,30] by direct and/or indirect pathways. (Figure 1). Hence, the development of new agents for melanin inhibition, particularly natural substances, is of considerable interest in the pharmaceutical and cosmetic industries. Recently, a new perspective strategy for skin depigmentation involving stem cells including NSCs, has been described [28,29,30]. NSCs have been used as cell sources for cell-based therapy for several neurological diseases [31,32,33]. Interestingly, it has been reported that several types of stem cells release many soluble factors into the culture medium, termed the conditioned medium (CM) that are useful for the treatment of skin diseases, such as wrinkle prevention/reduction [34], wound healing [35] and skin whitening [36]. Some of the secreted factors in the CM are cytokines, chemokines, growth factors, metabolites, bioactive lipids and vesicles or exosomes that may function in an autocrine or paracrine manner [37,38,39]. The secreted factors from MSCs and NSCs may be present as cocktails and act in concert to inhibit melanin synthesis in skin. In previous studies, it has been reported that these bioactive molecules from adipose stem cell-derived CM negatively regulated melanin synthesis via the direct interaction with *TYR, TRP-1*, and *TRP-2* [40,41,42,43]. The secreted factors from human umbilical cord blood-derived CM significantly suppressed melanin synthesis via *MITF* degradation by ERK pathway activation [41]. However, it was recently reported that the signaling molecules Dickkopf-1 (DKK1) stimulated from a melanoma cell line treated by neural stem cells-conditioned medium (NSC-CM) indirectly inhibited pigment formation by decreasing the intracellular expression levels of TYR and other melanogenic enzymes as well as *MITF* [44] (Table 1). However, identification and characterization of the components within CM responsible for this effect remained to be studied.

In this review, we describe the isolation and culture of NSCs and NSC-CM preparation and the proteomic analysis of the proteins secreted from stem cells, as well as discuss the potentials of NSCs and their derivatives involving melanin inhibition and its mechanisms, as a new material for skin depigmentation.

## 2. Neural Stem Cell (NSC) Characteristics

### 2.1. NSCs—Isolation and Characterization

**Figure 2:**
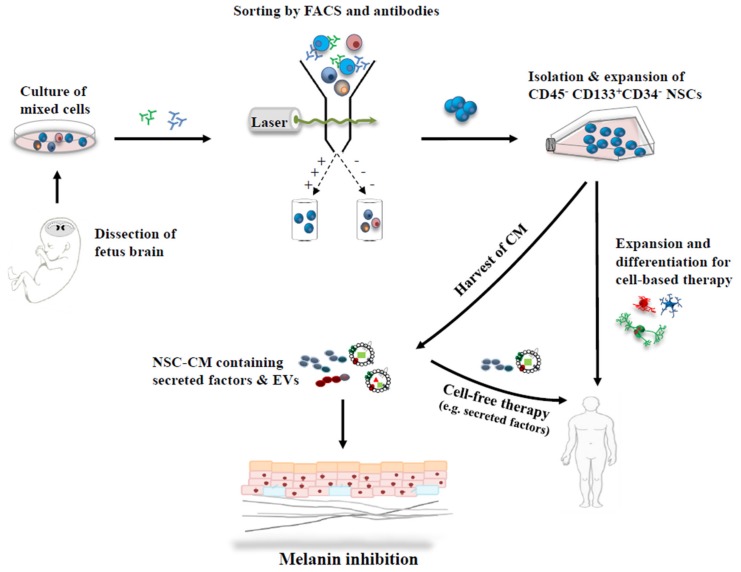
Strategic applications of stem cells and their derivatives, for skin depigmentation. CD45^−^/CD133^+^/CD34^−^ NSCs were isolated from the ventricular zone of human fetus brain by fluorescence-activated cell sorting and antibodies. NSCs transduced with retroviral vectors containing v-myc were expanded. They can differentiate into multiple lineages, neurons and glial cells, and can be applied to cell-based therapy and/or cell-free therapy using secreted factors. In addition, many factors including EVs are released in the medium during NSC cell culture, and they may modulate a microenvironment of skin tissue in a paracrine manner for melanin inhibition. FACS; fluorescence-activated cell sorting.

NSCs have the potential to differentiate into neuronal and glial cells, which are located in two germinal areas, the subventricular zone and the hippocampus in the mammalian brain [50,51]. In a previous study [52], CD45^−^/CD133^+^/CD34^−^ NSCs were isolated from the ventricular zone of 14-week gestational age, aborted human fetus, by fluorescence-activated cell sorting with monoclonal antibodies such as CD133, CD34 and CD45 [53,54] (Figure 2). The single NSCs were grown as neurospheres in 96-well plates with a culture medium containing N2 supplement, heparin (0.2 mg/mL), basic fibroblast growth factor (bFGF; 20 ng/mL), and leukemia inhibitory factor (10 ng/mL) [52,55]. Plates containing neurospheres were fed once a week depending on cell growth, and wells were evaluated for neuropsphere growth at 7–8 weeks. The neurosphere cells were established to human NSC lines, which were proven by molecular and cellular analysis using immunostaining assays with the NSC markers, such as nestin, SOX1 and musashi.

### 2.2. NSC—Culture and Expansion

Generally, human NSCs show a limited capacity for stable maintenance of the phenotype and karyotype during multiple passages [56]. Therefore, the immortalized human NSCs are feasible to overcome these limitations. The cell line can be steadily maintained during the long-term cell proliferation and the in vitro differentiation [56,57]. NSCs are cultured as monolayers on poly-ornithine and laminine/fibronectin coated tissue culture dishes. After approximately 14 days of cell culture at monolayers, NSCs are transduced with a retroviral vector containing v-myc. Several clones of human NSCs are isolated and expanded in Dulbecco’s modified Eagle’s medium (DMEM) supplemented with 10% fetal bovine serum, 100 U/mL penicillin, and 100 mg/mL streptomycin at 37 °C with 5% CO_2_. NSC clones express NSC-specific markers, and also give rise to neurons and glia after in vitro differentiation. Cytogenetic analyses of the NSC clones, at various passages, showed a normal human cell karyotype, without any chromosomal abnormality. The NSC clones are established as immortalized human NSC lines [58]. 

## 3. Melanin Inhibition with Conditioned Medium (CM) Derived from Neural Stem Cells

### 3.1. Conditioned Medium

The factors being secreted within a cell culture medium consist of secretomes and microvesicles including exosomes, and the medium is termed a CM [59]. The majority of the secreted factors, including growth factors, cytokines and chemokines, are released into the culture medium by fusion of secreted granules with the plasma membrane [60]. The bioactive molecules are also secreted and released via extracellular vesicles (EVs), which are classified into microvesicles and exosomes, according to their intracellular biogenesis pathway. This occurs by direct outward budding of the plasma membrane, in the case of microvesicles, whereas exosomes are formed within multivesicular bodies (MVBs) and secreted upon fusion of MVBs with the plasma membrane [61]. This multitude of secreted factors forms part of a complex network that provides amplification of regeneration and is largely influenced by the local microenvironment.

It has been proposed that the secreted factors could be beneficial to manipulate the microenvironmental states [62]. In addition, because the secreted factors derived from the CM can affect the activities of other cells within the local microenvironment, they have been considered as key mediators of paracrine activity [61].

For proteomic analysis, the CM was collected within 24 h after the NSCs were cultured in serum-free DMEM medium. The collected CM was briefly centrifuged and filtered using a 0.2-µm-pore-size syringe filter. Two different methods can be used to identify the secreted factors: the first is to use the human cytokine antibody array including 274 specific antibodies to detect the growth factors and cytokines simultaneously (RayBio^®^ Cytokine Antibody Array C Series). This method increases the reproducibility of proteomic profiling of the secreted factors including the small proteins and peptides. The targeted antibodies are presented in the pre-selection of the analytes. The second is to use shotgun proteomics analysis by liquid chromatography–tandem mass spectrometry (LC-MS/MS), which is currently a preferred approach and a sensitive technique, showing high throughput capability. However, it seems to be difficult to detect small proteins and peptides due to technical limitations. If the two methods are simultaneously applied to analyze the CM, a wide range of the various-sized secreted soluble factors can be examined and, subsequently, the valuable factors can also be defined.

### 3.2. NSC-Derived CM

NSCs can grow indefinitely and have a multipotent potential to differentiate into three major cell types of central nervous system: neurons, astrocytes and oligodendrocytes. Attempts have been made to use NSCs for cell-based therapy, as a means of regeneration of damaged brains, in many neurological disorders [31,32,33]. Cell transplantation allows introducing therapeutic cells to the damaged tissues with the ultimate aim of restoring the damaged tissues of the host. However, many studies showed that cell transplantation and subsequent differentiation at injury sites were very low and transient [63,64,65]. It has been reported that the therapeutic effects of stem cell transplantation can be exerted through the trophic factors secreted from the transplanted cells in the central nervous system [66], which suggests that the functional improvement of injured tissues afforded by stem cell transplantation could be amplified through the paracrine actions of the secreted factors. The transplantation studies, using NSCs, showed an increase in several secreted neurotrophic factors, such as brain-derived neurotrophic factor (BDNF), glial cell line-derived neurotrophic factor, and ciliary neurotrophic factor and, subsequently, resulted in neuroprotective effects in neurodegenerative disorders, such as spinal cord injury, ischemic stroke, Huntington’s disease, Parkinson’s disease, and demyelinating disorders [33,58,67,68,69]. In particular, NSCs are likely to exhibit the immunomodulatory effects as the MSCs have shown [70]. NSCs significantly decrease pro-inflammatory cytokines such as interleukin 2 (IL2), tumor necrosis factor α and interferon-γ as a suppressive effect on T cells [71], and can also interfere with the multiple inflammatory signals through the inhibition of T cell receptor-, IL2- and IL6-mediated immune cell activation and/or proliferation. These studies suggest that the diverse actions may underlie a broad anti-inflammatory effect of NSCs, which indicates relevance of NSCs and/or their derivatives to the various dermatological and cosmetic applications. It was reported that the CM consisted of the various growth factors by proteomic analysis [72,73]. Moreover, treatments using CM derived from the various types of stem cells has directly contributed to the recovery of the damaged tissues, rather than the stem cells themselves [74,75,76].

In our previous study [30], a melanoma cell line was treated with NSC-CM to decrease the expression of *MITF* and melanogenic enzymes. *MITF* is one of the nuclear targets of various signaling pathways, such as the Wnt/β-catenin, α-MSH and SCF pathways [77,78]. *MITF* regulates not only melanogenic enzymes but also melanocyte development, survival and proliferation [79]. Interestingly, the CM treatments influenced the morphological changes and growth rate of the melanoma cell line, which prompted us to determine that the Wnt/β-catenin pathway is associated with depigmentation and cell growth [80]. The inhibitors of the Wnt/β-catenin pathway were not identified in the NSC-CM, while expression of Wnt inhibitor *DKK1* in the target cell line was dramatically increased in vitro [30]. When melanoma cell lines were directly treated with recombinant human DKK1 proteins, melanin production was robustly suppressed [81,82]. Therefore, our group demonstrated that NSC-CM could inhibit melanin synthesis by disrupting the Wnt/β-catenin pathway via upregulation of endogenous *DKK1* expression [81]. In previous studies, it has been reported that *DKK1* as an inhibitor of Wnt/*β*-catenin pathway could be positively regulated by 1α,25-dihydroxyvitamin D3 [1,25(OH)2D3], the most active vitamin D metabolite, in various cancer cells [83,84,85]. However, 1α,25-dihydroxyvitamin D3 was not reported its presence in CM and its role in inhibition of melanin production, suggesting that it might be further studied in the future. In addition, another group reported that the secretion of cytokines TGF-β1, IL-6 and TNFα was detected in an adipose stem cell (ASC)-derived CM using an enzyme-linked immunosorbent assay (ELISA) [28], which, subsequently, contributed to the inhibition of pigment formation by downregulation of tyrosinase and TRP-1 proteins that were mainly mediated by TGF-β1 [28].

Using a human cytokine array, analysis of the NSC-CM components revealed that more than 30 proteins were highly expressed and secreted as follow: seven cytokines, four chemokines, five interleukins, five growth factors, five enzymes/inhibitors, two receptors and two adhesion molecules (data were not published). However, the concentrations of the secreted components were not measured by ELISA. Tachida et al. reported the secreted profiles of MSCs derived from bone marrow, adipose tissue and dental pulp [86] because of the increasing importance of MSCs showing the therapeutic effects in various diseases, such as cardiovascular disease [87], diabetes [88], neurodegenerative and inflammatory diseases [89]. They found 124 secreted proteins commonly produced among all three MSCs. Among them, the factors whose functions are involved in tissue regeneration, such as angiogenesis, migration, and inflammatory response, were CTGF, SERPINE1, TGFB1, DKK3, and MYDGF. The newly identified factors whose roles are not well characterized were AIMP1, CLEC11A, GAS6, HFGF, INHBA, and PCSK5. These factors could be contributed to explain the various therapeutic effects of MSCs [86].

Therefore, further investigation of the secreted factors identified by cytokine arrays of NSC-CM, or the proteomics analysis of NSC-CM, will be helpful to understand the mechanisms by which NSC-CM inhibits melanin synthesis.

### 3.3. Mechanisms of Melanin Inhibition by CM

**Figure 3:**
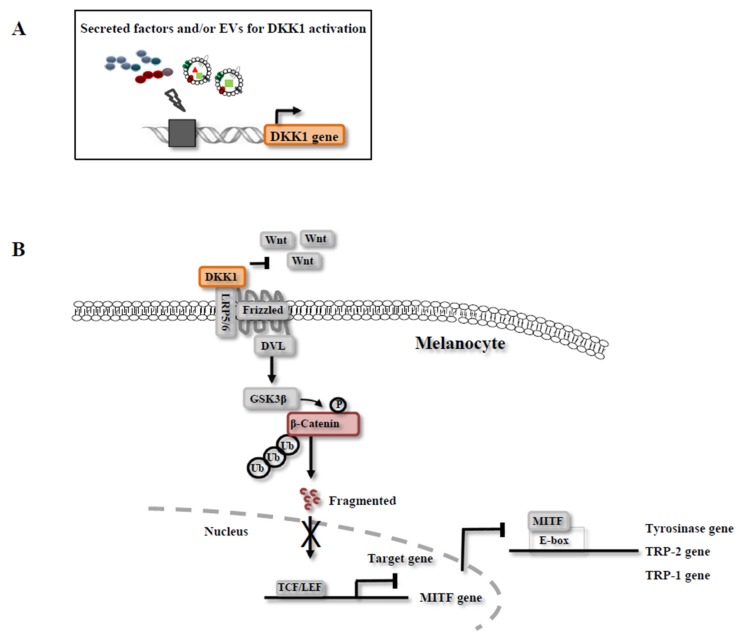
Signaling pathways for melanin inhibition by NSC-CM. The expression of Dickkopf-1 (*DKK1*) gene may directly or indirectly be induced by NSC-CM in melanocytes (**A**), which decreased the melanin synthesis via the inhibition of Wnt/β-catenin signaling pathway and subsequent downregulation of the expression of *MITF* and melanogenic enzymes (**B**). The thunder symbol indicates *DKK1* gene may directly or indirectly be induced by NSC-CM. TCF/LEF, T-cell factor/lymphoid enhancer factor*; MITF,* Microphthalmia-associated transcription factor; *DVL*, Dishevelled; * GSK3β*, Glycogen synthase kinase 3β; LRP5/6, Lipoprotein receptor-relased protein 5/6; Ub, Ubiquitination.

Our recent study showed that NSC-CM increased depigmentation via the inhibition of Wnt/β-catenin signaling pathway by triggering *DKK1* upregulation in a melanoma cell line and subsequent downregulation of the expression of *MITF* and melanogenic enzymes [30,90]. *DKK1* mRNA is not expressed in human adult tissues, except human placenta, while *DKK3* mRNA is found in many human adult tissues, such as heart, brain, and spinal cord [91]. *DKK1* is the only known secreted antagonist of Wnt signaling that interacts with transmembrane receptors [92,93], whereas other inhibitors of Wnt, including Wnt inhibitory factor-1 and secreted Frizzled-related proteins, directly bound to Wnt ligand to block the signaling pathways [94]. The skin on the palms and the soles is less pigmented than other areas of the body skin because palm fibroblast express high levels of *DKK1*. In addition, the expression of β-catenin in melanocytes cocultured with *DKK1*-transfected fibroblasts was decreased compared to melanocytes cocultured with control-transfected fibroblasts [43]. Therefore, the inhibitors of Wnt/β-catenin signaling may decrease the melanin production through β-catenin–mediated inhibition of *MITF* activity, which, in turn, modulates the growth and differentiation of melanocytes (Figure 3). In the case of ASC-CM, the effects of pigmentation inhibition by downregulation of *tyrosinase* and *TRP-1* proteins were mainly mediated by TGF-β1, which was known to play an inhibitory role in tyrosinase synthesis and *MITF* expression [28]. However, the whitening action of ASC-CM was mediated by TGF-β1, but not involved with *MITF* [28]. These results suggest that the other factors secreted from ASCs might compensate for the downregulation of *MITF* expression. In addition, it was reported that IL-6, secreted from ASCs, decreased the expression of the *MITF* and *tyrosinase* proteins and, subsequently, inhibited the melanocyte proliferation and melanin synthesis [48].

Melanin production is also regulated by a number of signaling pathways and transcription factors. A well-known factor that can induce tyrosinase expression is α-MSH that binds to MC1R to activate adenylyl cyclase to produce cAMP [95], which activates cAMP-dependent kinase A (PKA)/CREB and the phosphoinositide 3-kinase/serine-threonine protein kinase Akt (also known as protein kinase B) or MAPK/ERK pathways [96] and increases the expression of melanocyte-specific *MITF* [97], regulating the expression of melanogenic enzymes such as *TYR, TRP-1* and *TRP-2*. A c-Kit and its ligand SCF (c-kit/SCF) are also involved in melanogenesis through transcriptional activation of *MITF* [98].

## 4. Exosomes in CM

Extracellular vesicles (EVs) are phospholipid membrane-bound vesicles isolated from cell culture supernatants and enclose bioactive factors, including cytosolic proteins, lipids and coding or noncoding RNAs and DNAs [99,100]. As mentioned above, EVs can be formed and released by budding from the cell plasma membrane of the cell (e.g., microvesicles), or generated inside and secreted when these compartments fuse with the plasma membrane (e.g., exosomes). Exosomes are smaller (40–120 nm in diameter) and more homogeneous in size than microvesicles (200–1000 nm in diameter) [38,101,102]. All EVs bear surface molecules that allow them to be targeted to recipient cells. Once attached to a target cell, EVs can induce signaling via receptor-ligand interaction, or can be internalized within the cell, or fuse with the target cell’s membrane, releasing their contents into its cytosol, thereby, altering the physiological state of the recipient cell. [103].

Exosomes were isolated from the various origins of stem cells, such as NSCs and bone marrow-, umbilical cord-, and adipose-derived stem cells [104,105,106,107,108,109]. The spherical structures are easily visualized in stem cell-derived CM by electron microscopy and also precipitated by ultracentrifugation. The presence of exosomes can be easily evidenced by the enrichment in plasma membrane phospholipid and via the possession of exosome-associated proteins, such as the late endosome-associated protein Alix and tumor susceptibility gene 101 (TSG101), as well as by Western blot using several of the surface markers including the tetraspans CD9, CD81 and CD63 [110,111]. Detailed analyses, performed by mass spectrometry, antibody array and microarray, showed that exosomes released by most cell types including stem cells carry a complex cargo of nucleic acids, proteins and lipids, with a plethora of unique gene products (http://www.exocarta.org/), proteins (>9769), mRNA (>3408), miRNAs (>2838) and lipid entries (>1116) [112,113,114]. In addition, exosomes contain intracellular signaling and cellular communication proteins, such as β-catenin, Wnt, notch ligands, IL-1β and TNF-α [115].

Recently, exosomes have been increasingly reported as a major remedy for stem cell secretion to support the regenerative capabilities of stem cells in tissue repair [116]. According to their origin, they are likely to fulfill different functions. It was reported that exosomes from adipose-derived stem cells were effective in degrading amyloid B peptide (Aβ) in an in vitro model of Alzheimer’s disease by secreted exosomes containing neprilysin, an enzyme [106,117]. In addition, the exosomes from bone marrow- and umbilical cord-derived stem cells inhibited the growth of U87MG glioblastoma cells in vitro, whereas those from adipose-derived stem cells promoted cell growth. These results suggest that the effects of stem cell-derived exosomes enhance the paradigm of the stem cell secreted paracrine factors.

A recent report showed that keratinocytes communicate with melanocytes through exosomes for pigment transfer from melanocytes. The ultraviolet B irradiation-treated exosomes, carrying microRNA miR-203 secreted from black keratinocytes, were detected in melanocytes and enhanced melanin synthesis by increasing both the expression and activity of melanosomal proteins tyrosinase, a key enzyme in melanin biosynthesis, and microphthalmia-associated transcription factor, a master transcriptional melanogenesis regulator [118]. Interestingly, Anderson’s group reported the functional protein contents of MSC and their exosomes [119]. When MSCs were exposed to ischemic tissue-stimulated conditions, several putative paracrine effectors of angiogenesis were present in MSC exosomes such as platelet derived growth factor, epidermal growth factor, fibroblast growth factor, and most notably nuclear factor-kappaB (NFkB) signaling pathway proteins [119]. Another group reported the protein contents of MSC-derived microvesicles [120], which have contributed to recovery of damaged tissues in animal disease models [121]. The potential microvesicle proteins were surface receptors (PDGFRB, EGFR, and PLAUR), signaling molecules (RAS, MAPK1, CDC42 and VAV2), cell adhesion (FN1, EZR, CD47, integrins, and LGALS1/3), and MSC associated antigens (CD9, CD63, CD81, CD109, CD248 and CD276) [119]. These results suggest that NSC-derived exosomal proteins could be identified, which may also modulate the melanin production in skin tissue based on our previous results [30].

## 5. Conclusions

Natural and synthetic compounds inhibiting pigmentation have played a role as direct inhibitors of the tyrosinase enzyme, which is critical for melanin synthesis. However, it was reported that some of the compounds showed side effects and problems in suppressing melanin production. Recently, neural stem cell (NSC)-derived CM has been applied as a new material to regulate melanin inhibition for skin whitening. The bioactive molecules within NSC-CM inhibited melanin production in vitro and in vivo. However, the identifications and functions of each factor within NSC-CM consisting of the secretomes and microvesicles including exosomes were not completely characterized. Hence, further studies identifying and characterizing the microvesicle proteins and the other components within NSC-CM by various analytical tools will allow us to understand the inhibitory mechanisms of the secreted factors. In summary, NSC-CM will play central roles in inhibiting melanin production as an alternative material for cosmetic and pharmaceutical applications in the future.

## Figures and Tables

**Table 1:** Factors inhibiting melanin production and their properties.

Source	Factor	Properties	Inhibition Effects	Advantages	Disadvantages	**Ref.**
Synthetic compounds	Deoxyarbutin	-Synthesized without every OH-group of arbutin -Reversible inhibition of tyrosinase activity	Strong	-A sustained depigmentation effect -Low cytotoxicity	-Thermolabile in aqueous solutions, where it decomposes to hydroquinone	[16,45]
	α-arbutin (α-glucosides of arbutin)	-Easily hydrolyzed to release hydroquinone	Strong	-Strong ability to inhibit tyrosinase	-N/A	[46]
	Magnesium l-ascorbyl-2-phosphate	-Inhibitor of tyrosinase	N/A	-Low adverse side-effects -Reduced cytotoxicity relative to hydroquinone	-Not tested in skin models	[47]
Natural compounds	Hydroquinone	-Most effective inhibitor of melanin synthesis by glutathione depletion, melanosome degradation and melanocyte damage -Good tyrosinase inhibitor	Strong	-Gold-standard of depigmentation	-Permanent depigmentation and exogenous ochronosis following long-term use -Banned by the European Committee (24th Dir 2006/6/EC)	[13]
	Arbutin	-Good tyrosinase inhibitor	Modest	-Low melanocyte cytotoxicity -Inhibition of melanin synthesis by competitive and reversible tyrosinase action	-Chemically unstable and can release hydroquinone, the potential toxicity	[15,26]
	Kojic acid	-Good tyrosinase inhibitor	Modest effect	-Acts as a free radical scavenger	-Causes allergies, such as irritant contact dermatitis	[17]
	Azelaic acid	-Competitive tyrosinase inhibitor	Weak	-No relevant side effects	-No induction of depigmentation on normal skin	[18]
	Ascorbic acid	-Good tyrosinase inhibitor	Modest	-Useful for health and beauty of skin-Good antioxidative, anti-inflammatory and photoprotective effects	-Instability and rapid oxidization in aqueous solution	[21]
Stem cells	Human adipose-derived stem cells (ADSCs or ASCs)	-Inhibition of melanin synthesis is mainly mediated by highly secreted TGF-β1-CM greatly improved the facial indexes, such as Erythema and melanin	N/A	-CM contains biological effectors that decrease melanin, such as TGF-β1, TNF-α and IL-6-IL-6 and *TNFα* concentrations are lower than the IC_50 _value for tyrosinase activity	-No decrease of *MITF* expression in mouse B16 melanoma cell line-No defined key factors for melanin inhibition	[28]
		-Melanin inhibition by highly secreted IL-6	N/A	-Inhibition of cell proliferation of mouse melanocytes tyrosinase-Decreased *MITF*, *TRP-1 *and *TRP-2 *expression in melanocytes	-No significant decrease of tyrosinase expression in human melanocytes -No defined key factors for melanin inhibition	[48]
	Human umbilical cord blood (hUCB)	-TGF-β1 was highly secreted, but the regulatory mechanism by which TGF-β1 causes depigmentation was not elucidated	N/A	-Proteasomal degradation of *MITF* in melan-a mouse melanocytes		tgf
	Human placental stem cells (hPSCs)	-Only the Melanin index of the hPSC-CM group was significantly high compared to the ASC-CM group -CM greatly improved the facial indexes, such as Erythema and melanin		-Melanin index of hPSC-CM was improved in clinical research	-hPSC-CM are not reported in vitro and in vivo	[49]
	Human neural stem cells (NSCs)	-Melanin inhibition by regulation of the gene and protein expression of *TYR, TRP-1, TRP-2 *and *MITF*, in mouse B16 melanoma cell line -Expression of *DKK1* was significantly increased in CM-treated cells	N/A	-Inhibition of cell proliferationin mouse melanoma cell line -Inhibition of melanin production through high expression of Wnt/β-catenin inhibitors	-No defined key factors for melanin inhibition	[30]

CM, conditioned medium; *MITF*, Microphthalmia-associated transcription factor; *DKK1,* Dickkopf-1*; TGF-β1,*Transforming growth factor-beta1; *TYR*, Tyrosinase; *TRP-2,* Tyrosinase related proteins-2; *TRP-1*, Tyrosinase related protein-1; N/A; Not available.
